# Resting state fMRI connectivity is sensitive to laminar connectional architecture in the human brain

**DOI:** 10.1186/s40708-021-00150-4

**Published:** 2022-01-17

**Authors:** Gopikrishna Deshpande, Yun Wang, Jennifer Robinson

**Affiliations:** 1 AU MRI Research Center, Department of Electrical & Computer Engineering, Auburn University, 560 Devall Dr, Suite 266D, Auburn, AL 36849 USA; 2 Department of Psychological Sciences, Auburn University, Auburn, AL USA; 3 Alabama Advanced Imaging Consortium, Birmingham, AL USA; 4 Center for Neuroscience, Auburn University, Auburn, AL USA; 5 Key Laboratory for Learning and Cognition, School of Psychology, Capital Normal University, Beijing, China; 6 Department of Psychiatry, National Institute of Mental Health and Neurosciences, Bangalore, India; 7 Centre for Brain Research, Indian Institute of Science, Bangalore, India; 8 Department of Psychiatry, Columbia University, New York, NY USA

**Keywords:** Cortical layers, HRF, Functional connectivity, Resting state fMRI, Blind deconvolution

## Abstract

Previous invasive studies indicate that human neocortical graymatter contains cytoarchitectonically distinct layers, with notable differences in their structural connectivity with the rest of the brain. Given recent improvements in the spatial resolution of anatomical and functional magnetic resonance imaging (fMRI), we hypothesize that resting state functional connectivity (FC) derived from fMRI is sensitive to layer-specific thalamo-cortical and cortico-cortical microcircuits. Using sub-millimeter resting state fMRI data obtained at 7 T, we found that: (1) FC between the entire thalamus and cortical layers I and VI was significantly stronger than between the thalamus and other layers. Furthermore, FC between somatosensory thalamus (ventral posterolateral nucleus, VPL) and layers IV, VI of the primary somatosensory cortex were stronger than with other layers; (2) Inter-hemispheric cortico-cortical FC between homologous regions in superficial layers (layers I–III) was stronger compared to deep layers (layers V–VI). These findings are in agreement with structural connections inferred from previous invasive studies that showed that: (i) M-type neurons in the entire thalamus project to layer-I; (ii) Pyramidal neurons in layer-VI target all thalamic nuclei, (iii) C-type neurons in the VPL project to layer-IV and receive inputs from layer-VI of the primary somatosensory cortex, and (iv) 80% of collosal projecting neurons between homologous cortical regions connect superficial layers. Our results demonstrate for the first time that resting state fMRI is sensitive to structural connections between cortical layers (previously inferred through invasive studies), specifically in thalamo-cortical and cortico-cortical networks.

**Supplementary Information:**

The online version contains supplementary material available at 10.1186/s40708-021-00150-4.

## Introduction

The most distinct feature of the mammalian cerebral cortex is its laminar structure, comprised of cortical columns. A cortical column is a unit of complex information processing. It consists of processing chains that overlap, linking multiple inputs to multiple other outputs [1]. A single column of cerebral cortical gray matter normally has six layers. Different layers in the column have distinct distribution and types of neurons as well as separate connections with other cortical and subcortical regions. Our knowledge about cortical laminar-specific connections is mostly derived from invasive studies including histology, anatomical tract tracing, electrophysiology, and lesion methods [2–7], given that non-invasive modalities, such as magnetic resonance imaging (MRI), both anatomical and functional, have typically lacked the resolution to resolve layer-specific differences.

However, recent developments in ultra-high field functional MRI (fMRI) make it feasible to examine the blood oxygen level dependent (BOLD) signal from cortical and subcortical regions with sub-millimeter resolution. With such resolution, cortical layers can be resolved reasonably, although some amount of partial voluming still exists. In addition, reliable methods have been developed to obtain cortical parcellation in the native space of individual subjects [8–12]. In the recent past, laminar fMRI studies have investigated the spatial sensitivity of high-field fMRI to the neuronal response at the sub-millimeter level [12–21], primarily using activation paradigms [13, 15, 18, 19, 21–23]. The sensitivity of laminar fMRI has enabled us to understand the columnar profile of cortical activation at a finer spatial scale in the cerebral cortex. However, these investigations were only in the context of laminar fMRI activation (not resting state) within specific brain regions for specific stimuli (example: primary visual cortex with visual stimuli). In addition, previous results were most often achieved with partial brain coverage at ultra-high fields (7 T for humans and > 7 T in case of animal studies) unlike whole brain coverage used in conventional resting state fMRI studies.

One popular noninvasive method of analyzing cortical circuits at the voxel level is functional connectivity based on resting state fMRI [24]. Resting state functional connectivity (FC) has been shown to be sensitive to alterations in neural circuits in various mental disorders [25–30] as well as correlated with behavioral performance in healthy individuals [31–33]. Recent literature employing resting state fMRI based characterization of the human brain’s functional connectome suggests that resting state fMRI is grounded in underlying anatomical connections [34–37]. For example, simulations have shown that spatially distinct functional networks emerge in resting state data when they are constrained by the structural connectome [38, 39]. The close correspondence between functional and structural connectivity has also been confirmed with fMRI and diffusion tensor imaging (DTI) data [40]. This has been further confirmed in case reports of deficient inter-hemispheric functional connectivity in subjects with complete agenesis of the corpus callosum [41]. However, it is noteworthy that resting state functional connectivity can be sensitive to multi-synaptic interactions, and hence, regions that are not directly connected structurally could still be functionally connected. These data suggest that if two regions have a direct structural connection, then they should also be functionally connected, but the opposite may not be true. Consequently, one could expect a strong functional connection between regions that are also directly connected structurally. In this work, our objective is to extend this concept from mesoscale connections between brain regions to rather microscale connections between different cortical layers in these regions. Attempts to do so have been scarce in the literature. Below, we present previous attempts in this direction. Layer-specific connections between the primary visual cortex layers II/III and middle temporal area layer IV were detected with high-resolution resting state fMRI through functional connectivity analysis [20]. The default mode network under resting state was clearly seen across six layers by seed-based functional connectivity analysis after removing depth-dependent physiological noise [42]. In addition, a recent study showed the existence of temporal correlation of resting state hemodynamic signals derived from optical imaging at sub-millimeter columnar scale in the visual cortex [43]. These studies suggest that functional connectivity could be a potentially useful method to investigate the laminar connectional architecture at the functional level. However, it is yet unclear whether resting state functional connectivity (FC) is in fact stronger along structural pathways that connect different layers of brain regions compared to say, other possible connections between layers that do not have a direct structural projection between them. While this question has been addressed at the meso-scale voxel level, it has not been investigated at the micro-scale layer level. To test these possibilities, many technical challenges need to be surmounted as we discuss below, to provide motivation for the methodological choices we have made.

The major limitation of fMRI is that it is an indirect measure of neural activity, because it measures changes in blood oxygenation level that is modulated by the local vascular distribution (vessel size) and the activation-induced hemodynamic changes [21]. The BOLD fMRI signal can be modeled as the result of the convolution of a latent neural response and the hemodynamic response function (HRF). At the voxel level, the HRF varies across brain regions as well as across individuals [44, 45]. Some animal and human MRI studies at high field have shown that the response height and time-to-peak (TTP) of the HRF varies with cortical depth [18, 46–50]. It was shown that the deeper layers have faster and narrower hemodynamic response compared to the superficial layers. In addition, at the laminar level, gradient-echo BOLD signals have relatively poor laminar specificity, because they are more sensitive to larger vessels [51]. However, a recent investigation of the spatial point spread function (PSF) for the BOLD response showed that the laminar PSF of the gradient-echo BOLD signal had a “flat-tail” characteristic across layers, with the tail running to the pial surface [52]. This indicates that lower layers contribute signal to any given layer in gradient-echo BOLD. While spin echo BOLD may provide better spatial laminar specificity, one may lose sensitivity to the BOLD effect when using spin echo. Investigations into the laminar specificity of BOLD as well as HRF variability across cortical layers have invariably used task-based paradigms and cannot be readily generalized to resting state given that neurovascular coupling likely operates under a different regime in resting state (see extension of Buxton’s balloon model to resting state conditions [53].

Many studies have characterized the effect of HRF variability across regions and subjects [45, 54], as well as the impact of HRF variability across layers [18, 46–50]. However, all of these studies investigated the impact of HRF variability in the context of detecting activation (and not in the context of characterizing functional connectivity). Furthermore, inter-subject and spatial variability of the HRF could potentially give rise to a scenario, wherein the BOLD fMRI time series from any given two regions are synchronized, while the underlying neural response is not, thus giving high correlation between BOLD signals, while the true correlation between latent neural variables may be low. The opposite scenario, wherein the underlying neuronal variables are synchronized, while the BOLD fMRI time series are not, is also equally possible (see Additional file 1: Fig. S1 for illustration of these scenarios). Therefore, we need to extract the underlying latent neural response to get reliable estimates of FC between layers of different regions. The readers are referred to Rangaprakash et al. for more details on the effects of HRF variability on functional connectivity [55].

**Fig. 1:**
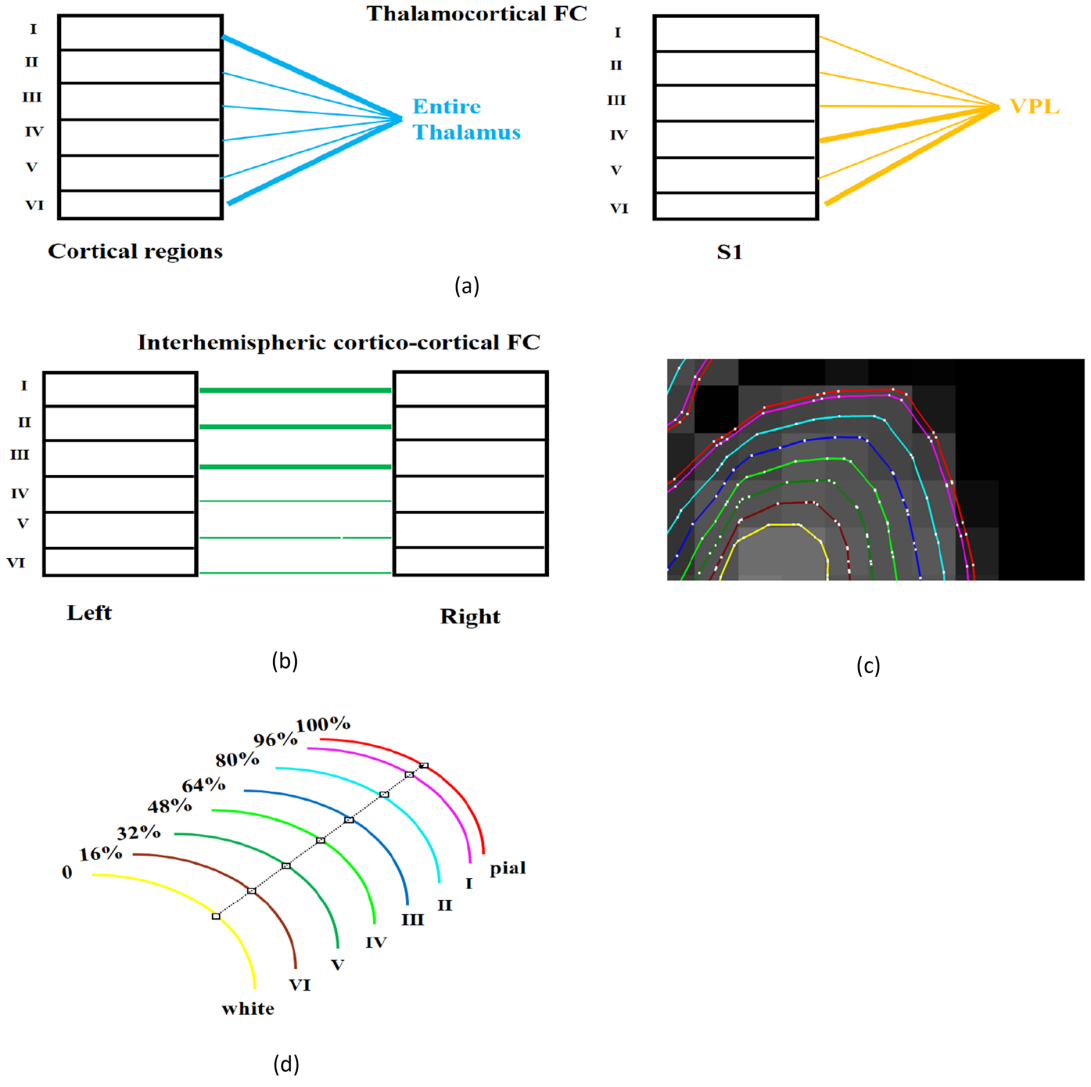
Illustration of our functional hypotheses that were motivated by previous invasive anatomical tract tracing studies. The width of the lines represent the strength of the connections. **a** Thamalocortical hypotheses: we hypothesized that FC between the entire thalamus and cortical layers I and VI will be significantly stronger than between the thalamus and other layers (blue, left panel). Furthermore, FC between somatosensory thalamus (ventral posterolateral nucleus, VPL) and layers IV, VI of the primary somatosensory cortex (S1) will be stronger than with other layers (yellow, right panel). **b** Cortico-cortical hypothesis: inter-hemispheric cortico-cortical FC between homologous regions in superficial layers (layers I–III) will be stronger compared to that in deeper layers (layers V–VI). **c** 6 surfaces plus white matter and pial surface overlayed on anatomical MRI (white matter surface: yellow, layer VI surface: brown, layer V: green, layer IV: lime, layer III: blue, layer II:, cyan, layer I: purple, and pial surface: red; the white dots are the vertices on these surfaces). **d** Illustration of the relative distance of 6 intermediate surfaces to white matter surface

In this study, we applied a surface-based laminar analysis pipeline available in FreeSurfer (https://surfer.nmr.mgh.harvard.edu/) to process high-resolution anatomical data with a 0.6 mm isotropic resolution and to delineate the six layers of the cortex [56, 57]. To investigate whether FC is sensitive to layer-specific connectional architecture, we examined this aspect with high-resolution resting state fMRI data (voxels with 0.85 mm in-plane resolution) obtained at 7 T. A simple blind deconvolution technique [58] was used to obtain the latent neural signals for each layer. Specifically, we tested the following hypotheses regarding thalamo-cortical and cortico-cortical layer-specific microcircuits derived from previous invasive anatomical studies (Fig. 1): (1) FC between the entire thalamus and cortical layers I and VI must be significantly greater than between the whole thalamus and other layers. This follows from evidence in rat brain tracing studies which show that regions across the cortex receive inputs to layer I from M-type thalamic neurons distributed in most thalamic nuclei [59–64]. In addition, pyramidal neurons in layer-VI are known to target all thalamic nuclei. Furthermore, FC between somatosensory thalamus (ventral posterolateral nucleus, VPL) and layers IV and VI of the primary somatosensory cortex (S1), must be stronger than other layers. This follows from the well-known C-type thalamic neurons in VPL that primarily target layer IV in the primary somatosensory cortex, and then corticothalamic pyramidal neurons in layer VI are known to project back to C-type thalamic neurons in VPL [65, 66], (2) inter-hemispheric cortico-cortical FC (i.e., between the left and right brain regions of the same area) in superficial layers (layers I–III) must be higher compared to deep layers (layers V–VI). This follows from evidence in rodents that 80% of the cell bodies of those callosal projecting neurons are distributed in layer II and layer III, with only 20% in layers V and VI [67, 68]. Other studies have claimed that layers I through III are the main target of inter-hemispheric cortico-cortical afferents, while some suggest that layer III is the main source of cortico-cortical efferents [69–71]. Taken together, it makes sense to hypothesize higher cortico-cortical FC in superficial layers compared to deeper layers.

We found that resting state functional connectivity at the laminar level, to a great extent, were in sync with the hypotheses stated above. To the best of our knowledge, we are the first to show that fine-grained layer-specific thalamo-cortical and cortico-cortical anatomical connections between cortical layers are reflected by stronger resting state functional connectivity in these pathways.

## Results

### Surface-based laminar analysis and blind deconvolution

**Fig. 2:**
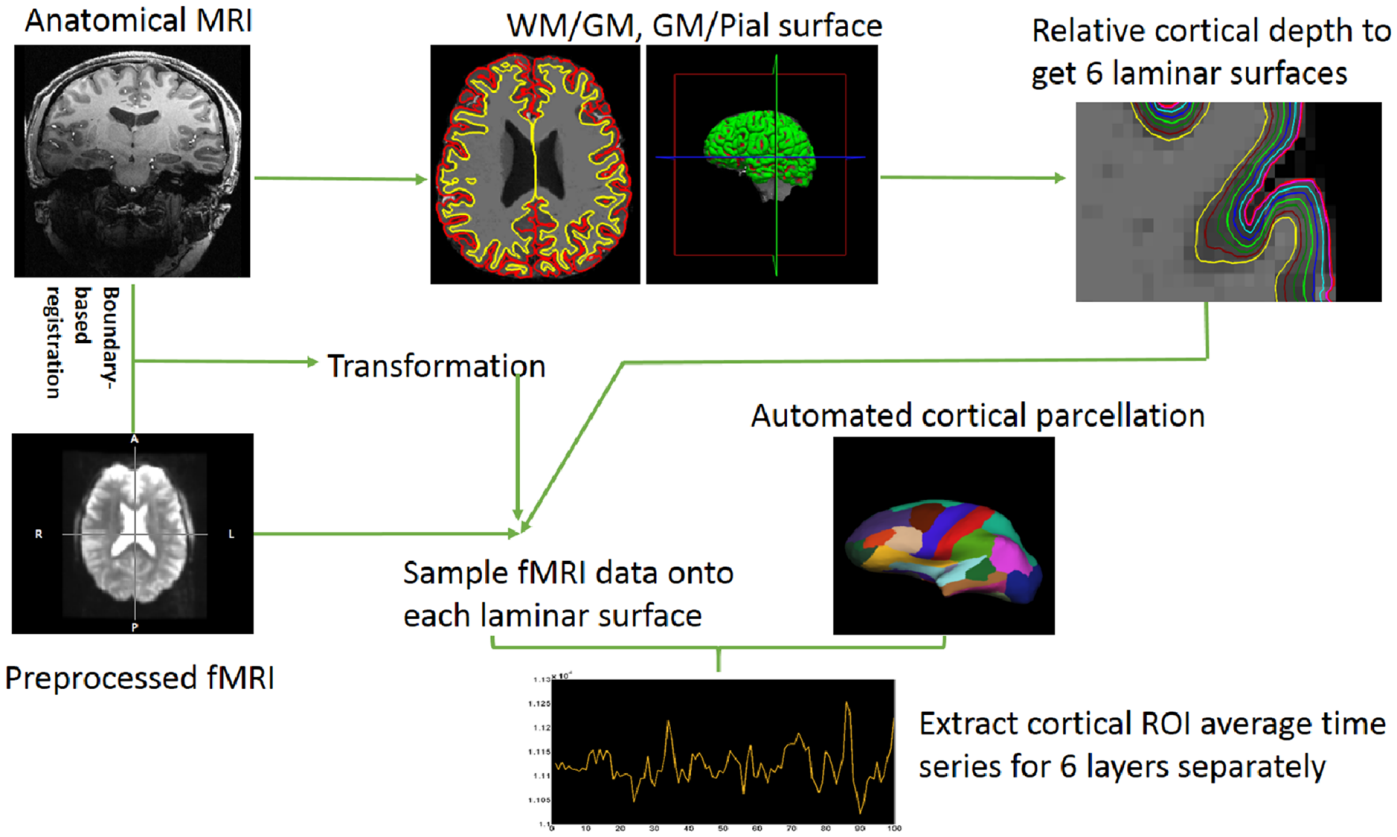
Schematic illustrating the laminar analysis pipeline for extracting mean time series from the six cortical layers for all 68 brain regions in the Desikan–Killiany atlas [9]

We employed a surface-based laminar analysis pipeline to extract vertex-based resting state fMRI time series for 68 cortical regions separately from six layers of the neocortex (Fig. 2). Then, we performed vertex-by-vertex blind deconvolution [58] to get each vertex’s latent neural response and HRF. Please refer to the methods section for further details on these analyses.

### Functional connectivity across cortical regions between layers

**Fig. 3:**
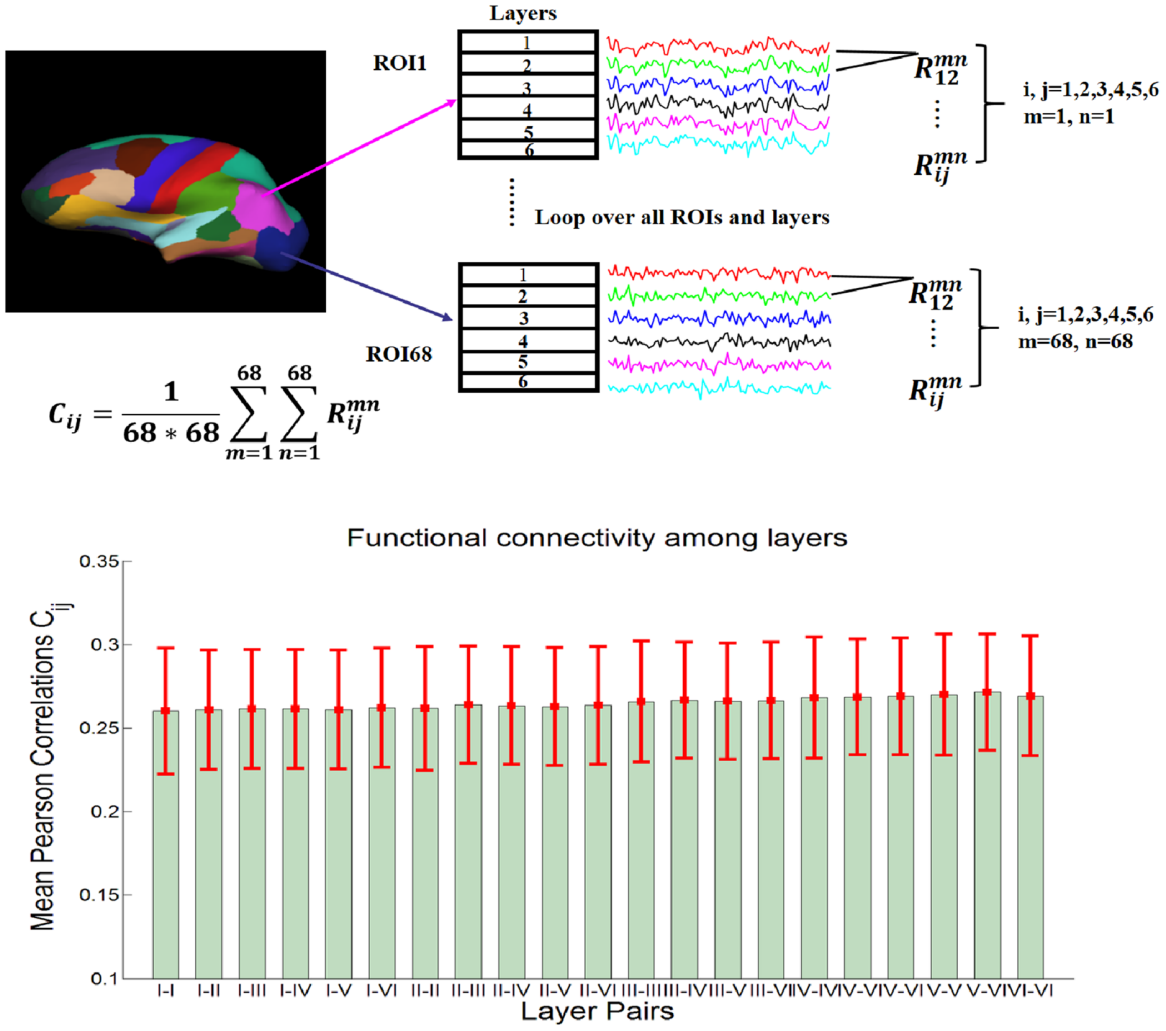
Top: an illustration of the method for calculating FC between all layers across all cortical regions to investigate global trends (*i*, *j* represents layer number; *m*, *n* represents regions, and *C*_*ij*_ represents the mean Pearson’s correlation between two given layers calculated across all cortical regions). Bottom: the mean Pearson’s correlation values between a given layer and all layers across all cortical regions in the Desikan–Killiany [9] atlas. No significant differences were found. 21 pairs are included. The error bar indicates the calculated standard deviation

With each vertex’s latent neural response, we calculated the mean time series for 68 regions of interest in each layer. To investigate global trends, we estimated the mean Pearson’s correlation between the 68 ROIs (using latent neural signals) in a given layer and those in all layers. The mean correlation did not show any significant difference between layers (Fig. 3). This demonstrates that global connectivity differences between layers were absent.

### Hypotheses testing before deconvolution

**Fig. 4:**
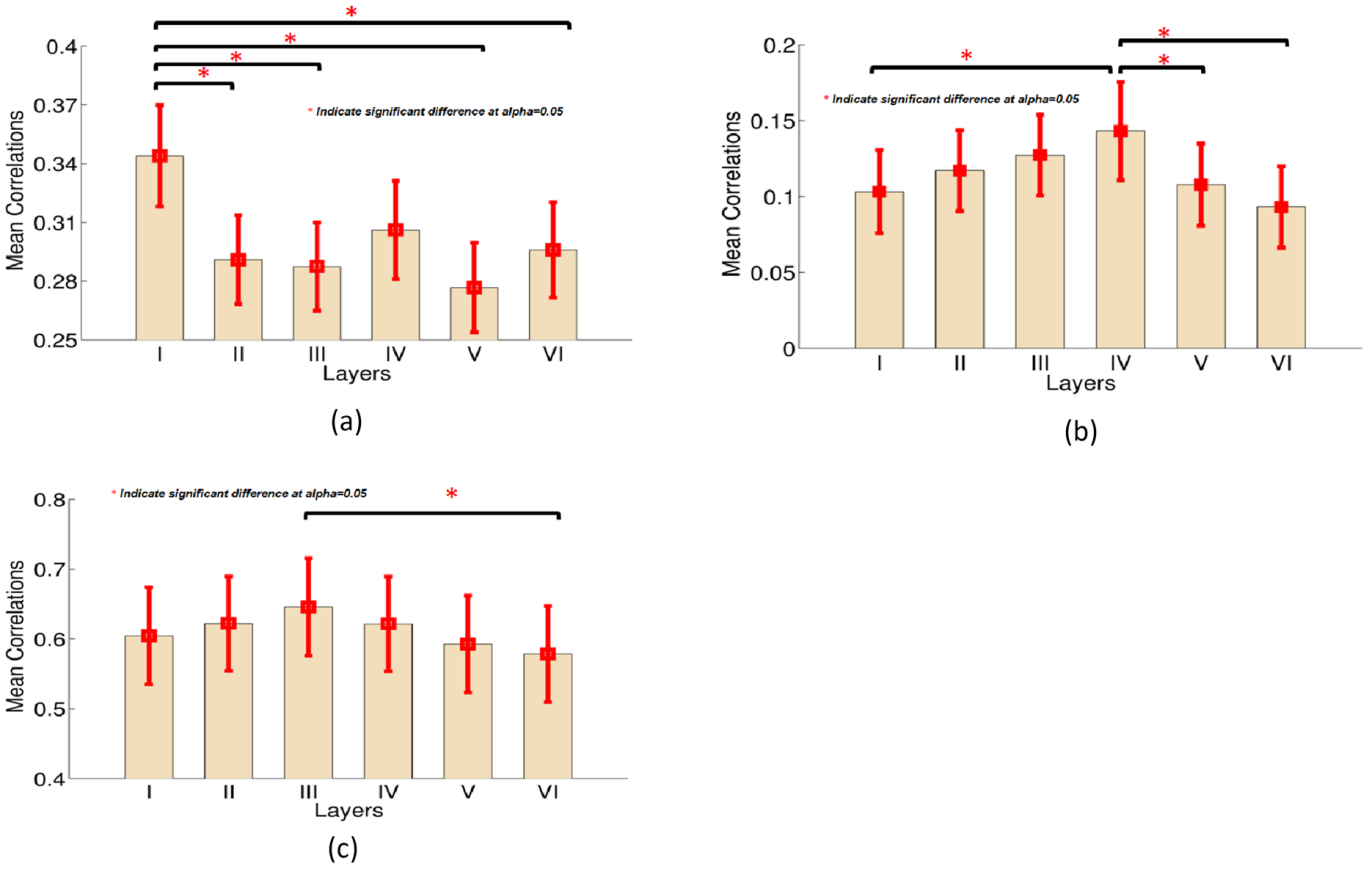
**a** Mean thalamo-cortical FC values between the entire thalamus and all cortical layers estimated from BOLD data before blind deconvolution; **b** mean FC between the somatosensory thalamus (VPL) and six different layers of primary somatosensory cortex before blind deconvolution; **c** mean inter-hemispheric cortico-cortical laminar FC values estimated before blind deconvolution. *Significant difference with *p* (corrected) < 0.05, the error bar indicates the estimated standard deviation

To test the thalamocotical hypothesis, we computed the Pearson’s correlation between the mean time series extracted from 68 ROIs in each layer with the mean time series extracted from the entire thalamus. For testing the specific VPL ↔ S1 connectivity hypothesis, the Pearson’s correlation between the mean time series from primary somatosensory cortex (S1) and the VPL were computed. For testing the cortico-cortical hypothesis, we estimated the mean inter-hemispheric correlations only between homologous regions in each layer. The functional connectivity pattern for thalamo-cortical connections showed that the mean Pearson’s correlation between layer I and the entire thalamus was strongest across the cortex (Fig. 4a), and was significantly (FDR corrected *p* < 0.05) greater than the correlation between the thalamus and layers II–VI. Although layer IV showed a trend to be more strongly connected to the thalamus, it did not reach significance. In contrast, VPL ↔ S1 connectivity was significantly stronger in layer IV than in layers I, V, and VI (Fig. 4b). We then examined the inter-hemispheric cortico-cortical connections for all 68 cortical regions for each layer (i.e., between the left and right brain regions of the same area). We found that the inter-hemispheric cortico-cortical mean correlation for layer III was significantly greater than layer VI (Fig. 4c).

### Laminar HRF differences

**Fig. 5:**
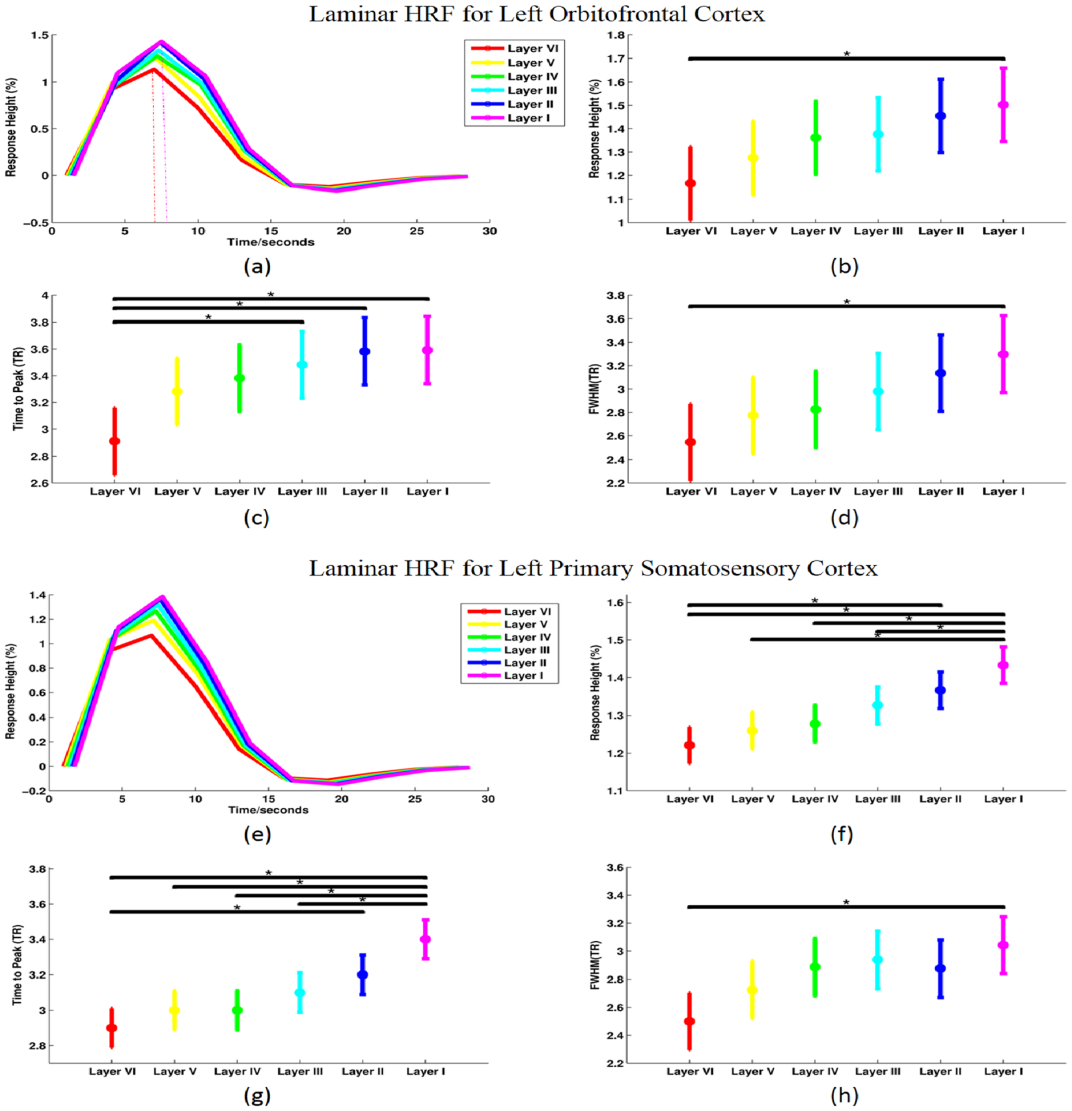
Region-specific HRF plot and multiple comparisons across the layers for left orbitofrontal cortex (OFC) (**a**–**d**) and left primary somatosensory cortex (S1) (**e**, **f**). The mean left OFC (**a**) and left S1 (**e**) HRF plot for six layers separately. Layer VI (red), layer V (yellow), layer IV (green), layer III (cyan), layer II (blue), and layer I (purple); multiple comparisons across the layers for left OFC (**b**) and left S1 (**f**) for response height; time to peak multiple comparisons across layers for left OFC (**c**) and left S1 (**g**); FWHM multiple comparisons across the layers for left OFC (**d**) and left S1 (**h**). *Significant difference with *p* (corrected) < 0.05. The error bar indicates the estimated standard deviation

The hemodynamic response could be different between regions across subjects and it has been previously shown that this might impact the estimates of connectivity obtained between such regions [44, 54]. To assess the laminar variability of the HRF and recover the neural response, we performed blind deconvolution before functional connectivity analysis. To assess the effect of deconvolution, we compared the shape of region-specific HRFs across six layers (Fig. 5). Three parameters of region-specific HRFs we examined were response height, time-to-peak, and full-width at half-max (FWHM). The means and standard deviations of the three parameters were calculated separately for each layer across all subjects. As an illustration, we show the region-specific HRF results for left orbitofrontal cortex (Fig. 5a–d) and primary somatosensory cortex (Fig. 5e–h), which are two of the 68 parcellation regions.

After one-way analysis of variance (ANOVA) for response height (*p* = 0.0469), time-to-peak (*p* = 0.0026), and FWHM (*p* = 0.0268) of region-specific HRF separately, we found response height as well as time to peak and FWHM were significantly different across the layers (*p* < 0.05 FDR corrected) for left orbitofrontal cortex. In addition, the three parameters were distinct across layers for left primary somatosensory cortex as well (*p* = 0.0035 for response height, *p* = 0.0014 for time-to-peak, and *p* = 0.0312 for FWHM). Furthermore, multiple comparison of means in one-way ANOVA was employed for each parameter. Here, for the left orbitofrontal cortex, we found that the response height and FWHM of layer I was significantly (*p* < 0.05 corrected) larger than for layer VI, and time to peak of layers I, II, and III was significantly (*p* < 0.05 corrected) larger than layer VI. For the primary somatosensory cortex, we found that the response height and time to peak of layer I were significantly (*p* < 0.05 corrected) larger than for layers III–VI, and at the same time, the response height and time to peak of layer VI were significantly smaller (*p* < 0.05 corrected) than layer II. Moreover, FWHM of layer I was significantly (*p* < 0.05 corrected) wider than layer VI.

**Fig. 6:**
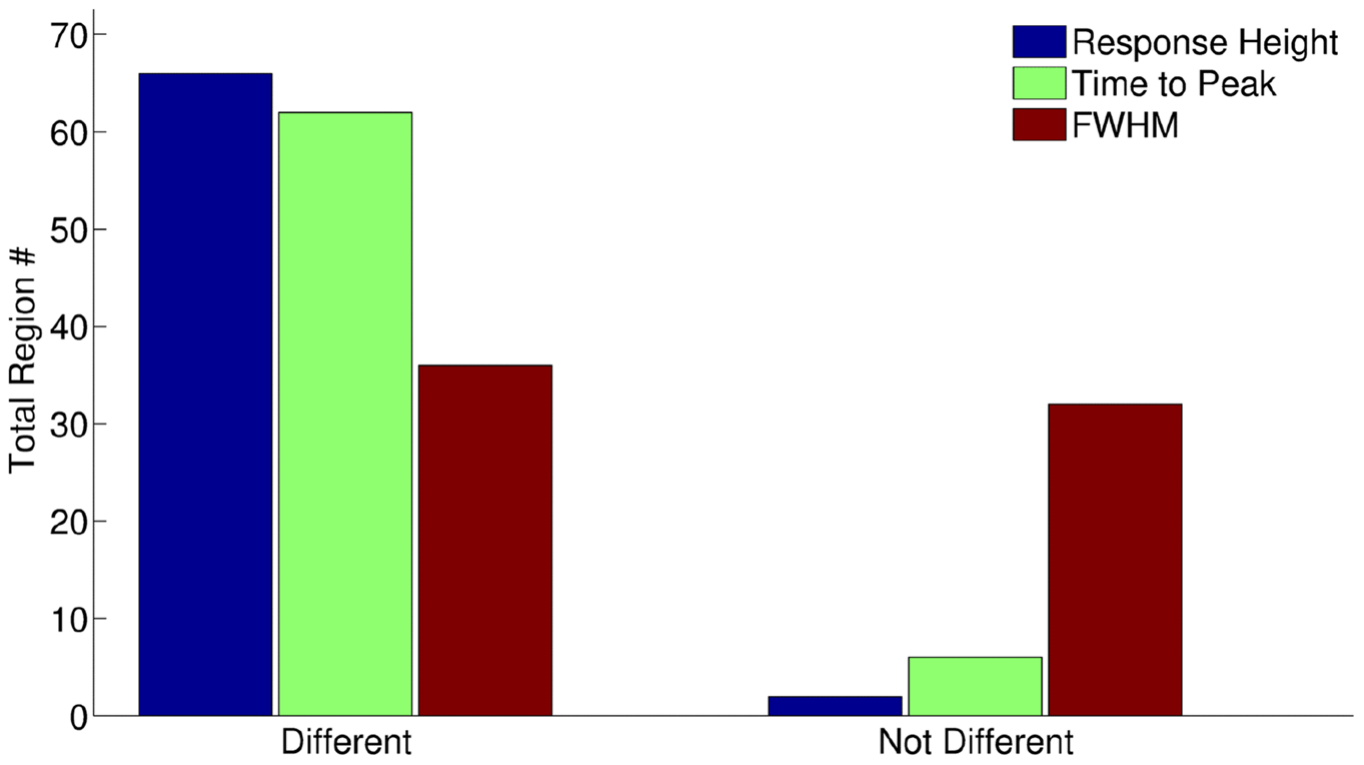
Summary of one-way ANOVA analysis performed on HRF parameters (response height, time to peak, and FWHM) for 68 regions. 66 out of 68 region had significant difference across the layers for the response height, 62 out of 68 regions for time to peak, and 36 regions for FWHM at p (corrected) < 0.05

To investigate whether this is a general fact for all region-specific laminar HRFs, we performed similar analyses for all other 66 regions and summarized the results in Fig. 6. 66 out of 68 regions had significant difference (*p* < 0.05 corrected) across the layers for the response height, 62 out of 68 regions for time to peak, and 36 regions for FWHM. In summary, the HRF varies across cortical layers in many brain regions and it is necessary to recover the latent neural signal at each layer before performing connectivity analysis.

### Individual-level FC difference before and after deconvolution

**Fig. 7:**
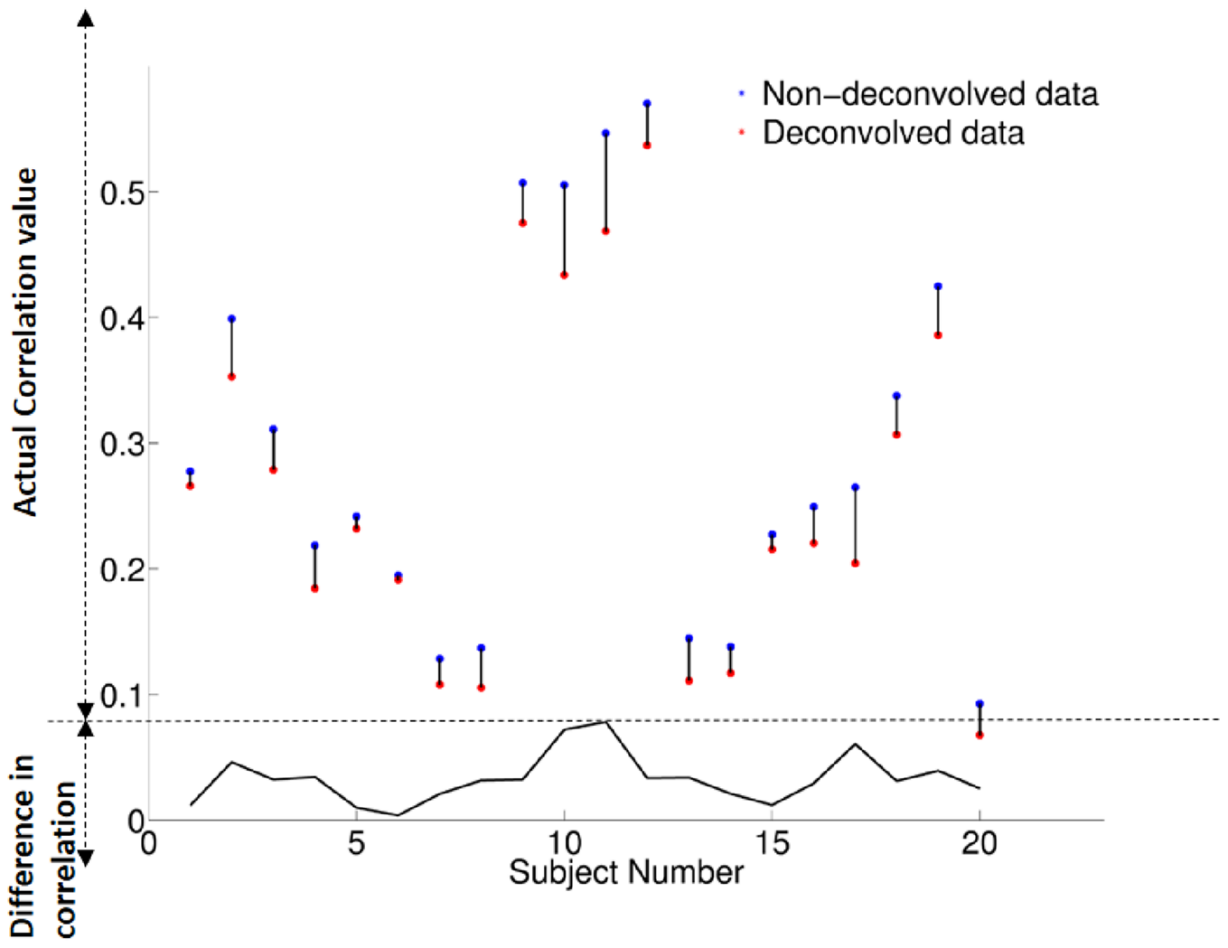
Comparison of the individual-level average FC values before and after deconvolution for all paths. Blue represents FC values with non-deconvolved data, and red for FC values after deconvolution

We estimated individual-level mean connectivity values of all possible connectivity paths between the 68 ROIs and the results for all 20 subjects, obtained with both deconvolved and non-deconvolved data, are shown in Fig. 7. The differences in connectivity due to deconvolution are plotted at the bottom part of Fig. 7, showing the magnitude of change caused by HRF variability in each subject. The group average non-deconvolved FC value was 0.033 higher than deconvolved FC value. A paired *t* test between non-deconvolved FC and deconvolved FC returned a high statistical significance for all paths (*p* = 0.0012).

### Hypotheses testing after deconvolution

**Fig. 8:**
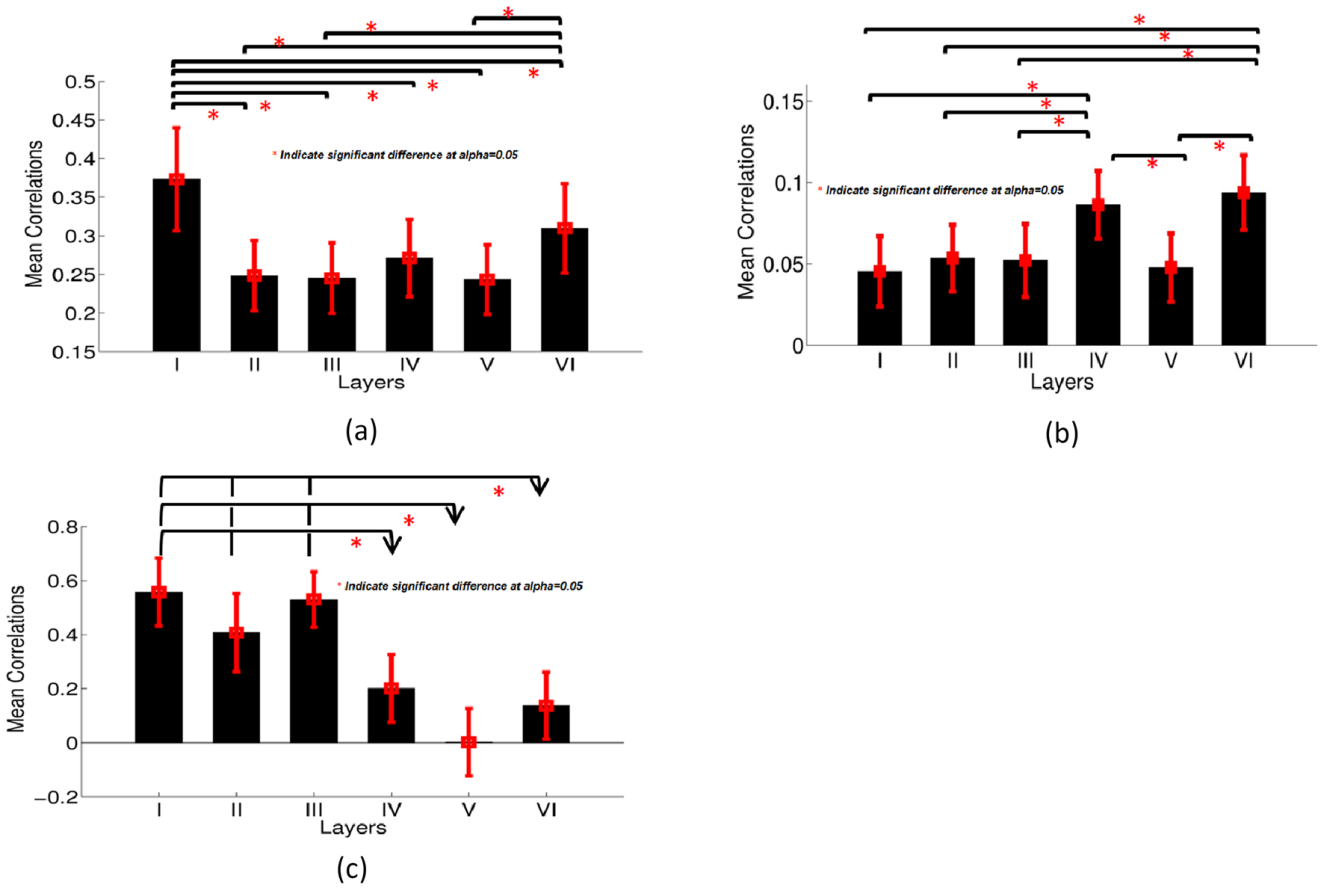
**a** Mean thalamo-cortical FC values between the entire thalamus and six cortical layers after blind deconvolution, i.e., using latent neural time series; **b** mean FC between the sensory core thalamus (VPL) and six different layers of primary somatosensory cortex after blind deconvolution; **c** mean inter-hemispheric cortico-cortical laminar FC values after blind deconvolution. *Significant difference with *p* (corrected) < 0.05. The error bar indicates the estimated standard deviation

Results obtained after deconvolution, i.e., those estimated from latent neural signals, were more in sync with our hypotheses. As we can see from Fig. 8a, FC between the entire thalamus and Layer I across the cortex was significantly greater than the FC between the entire thalamus and layers II–VI (FDR corrected *p* < 0.05). In addition, FC between the entire thalamus and Layer VI was significantly higher than FC between the entire thalamus and layers II, III, and V. In contrast, the FC between sensory core thalamus (VPL) and layers IV, VI of S1 was significantly stronger than between VPL and layers I, II, III, and V (Fig. 8b).

Finally, we examined the inter-hemispheric cortico-cortical FCs for each layer (i.e., between the left and right brain regions of the same area) and compared the FC results before deconvolution (Fig. 4c) with FC results after deconvolution (Fig. 8c). Before deconvolution, only the inter-hemispheric cortico-cortical FCs for layer III were significantly greater than layer VI (Fig. 4c). However, after deconvolution, FCs between homologous regions in layers I–III were significantly greater than in layers IV–VI (Fig. 8c). Generally speaking, the inter-hemispheric mean correlations in superficial layers were higher compared to those deeper layers [71].

## Discussion

In this study, we tested hypotheses involving thalamo-cortical and cortio-cortical layers specific microcircuits derived from previous invasive anatomical studies. The thalamo-cortical hypothesis is that FC between the entire thalamus and cortical layers I and VI must be significantly greater than that between the thalamus and other layers. This hypothesis is based on the fact that the regions across the cortex receive inputs to layer I from M-type thalamic neurons distributed in most nuclei of the thalamus and receive cortico-thalamic radiations from layer VI of the cortex [59–64]. Accordingly, we found that FC (estimated from latent neural variables) between the entire thalamus and layer I was indeed significantly greater than between the thalamus and layers II–VI, and FC between the thalamus and layer VI was higher than between the thalamus and layers II, III, and V. In addition, we found the FC between the sensory core thalamus (i.e., VPL) and layers IV and VI of the primary somatosensory cortex, were higher than other layers. This follows from the fact that C-type thalamic neurons in VPL primarily target layer IV in the primary somatosensory cortex, and then corticothalamic pyramidal neurons in layer VI project back to C-type thalamic neurons in VPL [65, 66]. To a large extent, the results confirmed our hypotheses. The cortico-cortical hypothesis is that inter-hemispheric cortico-cortical FC in superficial layers (layers I–III) must be higher compared to deep layers (layers V–VI) following evidence in rodents that 80% of the cell bodies of those callosal projecting neurons are distributed in layer II and layer III, with only 20% in layers V and VI [67–71]. Our results suggested that the inter-hemispheric FC was significantly higher in superficial layers than deeper layers. To our knowledge, this is the very first study to investigate the sensitivity of resting state fMRI connectivity at sub-millimeter spatial scale to the connectional architecture at the laminar level.

One common concern in laminar fMRI studies is the large signal amplitude on the pial surface, which could be potentially affected and contaminated by large veins on the cortical surface, or partial volume effects from large voxel sizes. Different methods have been employed to resolve the large vein problem. Simply avoiding the first layer compartment is the easiest way [21]. Indeed, if we did consider our hypotheses by excluding the first layer, they would be confirmed by our results. Another alternative approach is restricting the laminar analysis to strongly activated clusters in each subject [18]. A novel pial vein pattern analysis by optical imaging was suggested by Chen et al. to remove voxels associated with large veins, and the vein-free fMRI exhibited clear laminar specificity [13]. However, around 40% of activated voxels in the primary visual cortex was excluded for this study. In addition, optical imaging technique used by Chen et al. was invasive, and hence is not suitable for human studies. In this study, we approached this issue in terms of HRF differences across layers. We reasoned that any differences between BOLD signals across layers that have a vascular origin, must be reduced or eliminated if voxel (or vertex-specific) HRF was deconvolved from the BOLD data and connectivity estimation was performed in the latent neural space.

To investigate the variability of HRF across layers, we employed a simple but powerful blind deconvolution technique to recover the latent neural signal at each vertex. Our results showed that all three parameters of region-specific laminar HRF (response height, time-to-peak, and FWHM) varied in reference to cortical depths, and were significantly greater in superficial layers than deeper layers. This finding matches findings from previous HRF studies in animals. Tian et al. found both the onset of the BOLD response and the initial dip rely on cortical depth, and the fastest response was in deeper layers within the rat primary somatosensory cortex [49]. In addition, Yu et al. showed the onsets at different layers coincided with the neural inputs with line-scanning fMRI both in rat somatosensory cortex and motor cortex [47]. We have demonstrated that this is a general fact for almost all cortical regions. The comparison of functional connectivity before and after deconvolution showed the importance and necessity of recovering latent neural signals before any resting state functional connectivity analysis is performed at the laminar level. The functional connectivity post-deconvolution in the latent neural space aligned more closely with the underlying anatomical connections compared to FC obtained on BOLD data.

### Limitations and future work

There are a few limitations of present study, which need to be addressed in future layer-specific fMRI functional connectivity related research. First, different methods exist for identifying different cortical lamina from MRI data. The method we employed was to construct laminar profiles, which keeps a relatively fixed distance to the cortical boundaries (Fig. 1), the so-called equidistant laminae [18, 72, 73]. An alternate approach is the equipotentials method, wherein the equipotentials are computed between the inner white matter surface and pial surface with the Laplace equation, and then the cortical profiles can be constructed along the gradients [74]. However, the drawback with this approach is that the Laplacian equation may not match the anatomical layers observed from high-resolution MRI [75]. Recently a new model called equal-volume model for identifying cortical laminae was proposed by Waehnert et al. [75], and they claimed that it provides a better fit to observed cortical layering. In future, studies must compare the three different models for how well functional connectivity derived from layers constructed by them match the underlying anatomical predictions.

Second, the spatial laminar point spread function (SL-PSF) of the BOLD response presents a fundamental stumbling block for gaining laminar specificity in fMRI data. Lower layers always contribute signal to the upper layers, because the intracortical veins (ICV) are perpendicular to the surface, and the draining blood flows along the ICV into pial veins on the pial surface [52]. The interpolation-averaging method, wherein the fMRI volume is interpolated at certain cortical depths and the surface profiles are averaged, has been proposed for addressing this issue [18, 21, 22], but a more precise method to extract laminar signals is needed. As we briefly mentioned in the introduction, this is especially true for gradient echo EPI based fMRI which has a flatter PSF compared to spin-echo based EPI. Therefore, future studies may investigate whether spin echo EPI may be better for FC studies at the laminar level, even with the loss of sensitivity in spin echo compared to gradient echo. Recently, an extension of the Friston–Buxton hemodynamic model, which accounts for blood draining effects by coupling local hemodynamics across layers in dynamic causal models of fMRI during visual activation, was reported [14]. However, priors about two parameters controlling blood draining effects (the delay *τ*_*d*_ between the lower and upper layers, and *λ*_*d*_ which represents the strength of the blood draining effect from the lower to the upper layers) need further experimental validation in human resting state studies. Investigations into the laminar specificity of BOLD have invariably used task-based paradigms and cannot be readily generalized to resting state given that neurovascular coupling likely operates under a different regime in resting state (see extension of Buxton’s balloon model to resting state conditions [53]. Therefore, further modeling and experimental work is needed in this area, which could potentially lead us to a reliable and accurate laminar time series that will allow a more fine-grained investigation of resting state FC at the laminar level.

Third, the hypotheses we chose to test provide only an initial demonstration of the sensitivity of resting state fMRI functional connectivity to layer-specific functional microcircuits in the human brain. However, further fine-grained investigations are possible. This could involve specific thalamo-cortical pathways from other thalamic nuclei, specifically in systems that are unique in humans and for which we do not have reliable homologues in animals, and hence are not amenable to invasive investigations. For example, two parallel layer-specific pathways connect language-related thalamic nuclei to layer I and middle layers of Broca’s area. The cortico-thalamic radiations from Broca’s area in turn originate from cortical layers V and VI. Dysfunction in these pathways are important in aphasic patients with damage to the thalamic nuclei [76]. Our study opens the possibility of characterizing such layer-specific microcircuits, both in healthy and clinical populations, using ultra high field fMRI in the future.

Fourth, it is well recognized that functional connectivity cannot decipher the direction of information flow between regions, where as many anatomical projections which we have based our hypothesis on, are in fact directional in nature. Therefore, the next logical steps would be to test whether directional connectivity models of fMRI such as dynamic causal modeling (DCM) [77] and Granger causality (GC) [78–81] are sensitive to direction-specific anatomical projections at the layer-level.

Fifth, we have used the DK atlas to identify regions of interest given the fact that it is widely used and available as a surface. However, better atlases are available for volume-based analysis, which are less coarse and homogeneous than the DK atlas. Using such an atlas in the surface domain may improve the fidelity of the results.

Sixth, we do acknowledge that there may be regional specificity in the structure–function relationship, but previous evidence suggests that such specificity may be less for thalamo-cortical connections as well as inter-hemispheric connections between homologous regions. In addition, not all cortical regions have six layers. Therefore, it is reasonable to surmise that the broad nature of our hypotheses might have averaged out some effects and decreased their effect sizes. Testing more specific hypotheses in future studies may unravel the full extent of the utility of layer specific FC investigations.

Finally, we employed a gradient echo EPI sequence optimized for SNR and spatial resolution. The sequence used for data acquisition for testing FC-related hypotheses at the laminar level could well be optimized in other ways. This includes using a spin echo sequence and trading sensitivity for a narrow SL-PSF, as well as using a multiband EPI sequence to obtain a shorter TR, possibly at the cost of SNR (but not spatial resolution). One way of increasing the spatial resolution further would be to restrict coverage to regions specifically relevant to the hypothesis being tested, but this would require a custom processing pipeline (other than the one in FreeSurfer) that does not require whole brain coverage. In summary, our seminal study offers a lot of possibilities for investigating the brain’s functional connectome at a more fine-grained laminar spatial scale.

## Methods

### Data acquisition

Twenty healthy adult subjects (10 males, 10 females; 24.5 ± 3.3 years of age) participated in this study. All subjects provided informed consent, and the experimental protocols were approved by the Auburn University Institutional Review Board. High resolution resting state fMRI data was obtained from twenty healthy individuals using an EPI sequence with the following parameters: 37 slices acquired parallel to the AC–PC line, 0.85 mm × 0.85 mm × 1.5 mm voxels, TR/TE: 3,000/28 ms, 70º flip angle, base/phase resolution 234/100, A → P phase encode direction, iPAT GRAPPA acceleration factor = 3, interleaved acquisition, 100 timepoints. Data were acquired on a Siemens 7 T MAGNETOM outfitted with a 32-channel head coil by Nova Medical (Wilmington, MA). During resting state, the subjects were instructed to keep their head as still as possible, keep their eye open and let their mind wander and not think about anything specific.

A whole-brain high-resolution three-dimensional (3D) MPRAGE sequence (256 slices, 0.6 mm × 0.6 mm × 0.6 mm, TR/TE: 2,200/2.8, 7º flip angle, base/phase resolution 384/100%, collected in an ascending fashion, acquisition time = 14:06 min) was used to acquire anatomical data.

### Functional MRI data preprocessing

First five timepoints were discarded from the analysis to allow for MR equilibration. Slicing time correction was applied, and all functional MRI data were motion corrected using rigid body registration using SPM software (http://www.fil.ion.ucl.ac.uk/spm/). Next, linear trends were removed from each voxel time series. We also removed nuisance variance in the data by regressing out mean time series from ventricular CSF, white matter, as well as six head motion parameters. Importantly, spatial smoothing and spatial normalization were not performed. Spatial smoothing negates the advantages gained by smaller voxels sizes. In addition, spatial smoothing is employed in traditional general linear model based activation analysis to satisfy the assumptions of random field theory. We did not perform those kinds of analysis and hence found it unnecessary to spatially smooth the data. Next, the Freesurfer analysis pipeline enables individual-specific cortical parcellation from which we extracted the time series used in the analysis. Therefore, we found that spatially normalizing the data into a common space and incurring the costs of blurring and registration errors associated with such a procedure was unnecessary and may be counter-productive for the small voxel size we had and the type of analysis we planned.

### Surface-based MRI analysis

Cortical surface reconstruction of the cerebral cortex from magnetic resonance images is a major step in the quantitative analysis of the human brain structure. Cortical reconstruction approaches with Freesurfer are optimized for standard resolution (~ 1 mm) data. However, in this work, we applied Lüsebrink’s method to preprocess high-resolution anatomical MRI data with our original 0.6 mm isotropic resolution using FreeSurfer 6 beta version [82]. The white/gray and gray/CSF interfaces, as well as cortical thickness maps were automatically generated with FreeSurfer (Fig. 1). The surfaces generated by Freesurfer are represented in the form of triangular meshes, and each triangle has three vertices. In addition, a set of 3D coordinates of these surfaces gives the position of the vertices.

Once we obtained the white matter and pial surfaces, the laminar profiles were delineated within the cortical gray matter. They were constructed at fixed relative distance between the white matter and pial surfaces, determined from cortical thickness [21]. The position of each vertex on intermediate surfaces depends on the position of the correponding vertex on the white matter surface (Fig. 1). The first intermediate surface was located at 16% of cortical thickness away from the white matter surface, then at 32%, 48%, 64%, 80%, and 96% depths, giving us a total of 6 layers (Fig. 1). In addition, cortical regions defined on the inflated surface were automatically obtained from the Desikan–Killiany (DK) Atlas, including the primary somatosensory cortex [9]. The whole thalamus was identified in MRI volume data using automatic subcortical segmentation proposed by Fischl et al. [83]. We defined the sensory core thalamus (VPL) mask from the Oxford thalamic connectivity atlas [84] in common MNI space, and transformed the mask to each individual’s coordinate space.

### Registration of functional MRI to anatomical MRI

To enable the analysis of laminar fMRI, we need to align the fMRI volumes to those intermediate laminar surfaces. Apparently, the gray/white matter boundary in the EPI volume is easily identified automatically. We employed a method called boundary-based registration (BBR) [85]. It identified the interface between gray matter and white matter in the EPI data and then calculated a 12 degrees of freedom affine transformation, which registers the interface in EPI data to the corresponding surface reconstruction from the anatomical data (Fig. 2). After the registration, the results were visually inspected for each subject and manually edited, if needed [85].

### The extraction of functional MRI data from different layers

The preprocessed fMRI volume data were then transformed onto the six laminar surface reconstructions using the transformation matrix obtained in the previous step above. An average time series was extracted from the whole thalamus. This was done, because our first hypothesis involved the M-type thalamus cells distributed in each nucleus of thalamus [59–64]. Next, time series from each vertex in 34 lateral cortical ROIs in the DK atlas [9] were extracted, separately for left and right hemispheres in each subject. The 68 ROIs’ mean time series corresponding to the cortical ROIs were extracted for each of the 6 layers (Fig. 2). The regions in the DK atlas are defined on an inflated surface by manually tracing from the depth of one sulcus to another, thus incorporating the gyrus within. Therefore, unlike volume based ROIs that are affected by the folding pattern of sulci and gyri, the surface-based definition of DK atlas ROIs means that the results are not affected by the gyral and sulcal folding patterns.

### Blind deconvolution

After the time series in the preprocessed functional data were transformed onto the six laminar surface reconstructions, we performed vertex by vertex (a vertex is a point on a triangle surface as explained before, see Fig. 1c) blind deconvolution [58] to get each vertex’s latent neural response and HRF.

Hemodynamic deconvolution of the BOLD signal is under the assumption that the relationship between a latent neural signal and the BOLD response can be modeled as a linear and time invariant system, which can be described as follows:

1 $$ y\left( t \right) = x\left( t \right) \otimes h\left( t \right) + e\left( t \right) $$

where $y\left(t\right)$ denotes the observed BOLD signal, $x\left(t\right)$ denotes the underlying latent neural signal and $h\left(t\right)$ and $e(t)$ represent the HRF and the noise term in the measurement, respectively. Since the three terms in right side are unobservable quantities, we consider the neuron activity term $x\left(t\right)$ as a simple on–off model with series of delta functions $\hat{x}\left( t \right)$ as

2 $$ \hat{x}\left( t \right) = \mathop \sum \limits_{\tau = 0}^{\infty } \delta \left( {t - \tau } \right). $$

Note that the delta functions modeling the events exist at random times, which essentially amounts to modeling the resting state data as an event-related paradigm with randomly occurring events. Then the HRF $$h\left(t\right)$$ was fitted using a canonical HRF (two gamma functions) and two derivatives (temporal derivative and dispersion derivative). The parameters of $$h\left(t\right)$$ were allowed to vary for each time series. The approximation $$\overset{\lower0.5em\hbox{$\smash{\scriptscriptstyle\smile}$}}{x} \left( t \right)$$ of the latent neural signal can be obtained from the observed data using a Wiener filter as described below:

3 $$ \overset{\lower0.5em\hbox{$\smash{\scriptscriptstyle\smile}$}}{x} \left( t \right) = d\left( t \right) \otimes y\left( t \right), $$

where $\otimes$ denotes convolution. Applying Fourier transforms to $h(t)$, $y(t)$, $e(t)$, and $d(t)$, respectively, we get $H(\omega )$, $Y(\omega )$, $E(\omega )$, and $D(\omega )$. $D(\omega )$ can be expressed as follows:

4 $$ D\left( \omega \right) = \frac{{H^{*} \left( \omega \right)}}{{\left| {H\left( \omega \right)} \right|^{2} + \left| {E\left( \omega \right)} \right|^{2} }}, $$

where $$*$$ denotes complex conjugate. The estimate $$\overset{\lower0.5em\hbox{$\smash{\scriptscriptstyle\smile}$}}{x} \left( t \right)$$ of the latent neural signals $$x\left(t\right)$$ is then given by

5 $$ \overset{\lower0.5em\hbox{$\smash{\scriptscriptstyle\smile}$}}{x} \left( t \right) = {\mathcal{F}}^{ - 1} \left\{ {D\left( \omega \right)\left. {Y\left( \omega \right)} \right\} = {\mathcal{F}}^{ - 1} } \right.\left\{ {\frac{{H^{*} \left( \omega \right)Y\left( \omega \right)}}{{\left| {H\left( \omega \right)} \right|^{2} + \left| {E\left( \omega \right)} \right|^{2} }}} \right\}. $$

In Eq. 5, ${\mathcal{F}}^{-1}$ is the inverse Fourier transform operator. Since the measurement noise $e(t)$ is assumed to be white, the covariance of the noise term must be 0. For task-related fMRI, the stimulus function provides the prior information about neural activity and a generative model whose inversion corresponds to deconvolution. Here, resting state fMRI is considered as a spontaneous event-related signal, and these events can be reflected by relatively large amplitude BOLD signal peaks [58, 86]. Therefore, the time series from each vertex was evaluated against a given amplitude threshold around the local maximum (threshold was set to 1, since the input time series were normalized) to obtain a set of estimated onsets (the timing of delta functions) for these pseudo-events. To get the delay $\tau$ (the delay between the peak of fMRI and the peak of neural signal), we searched all integers between [0 8] based on a previous study that reported 4–8 s latencies in the gray matter [87]. Then the optimal integer was chosen as $\tau$ for which the covariance of noise $cov\left( {y\left( t \right) - \hat{x}\left( t \right) \otimes h\left( t \right)} \right)$ was smallest, to obtain the set of onsets. Subsequently, the vertex-by-vertex HRF was fitted and extracted with these pseudo-events. The readers are referred to Tagliazucchi et al. [86] and Wu et al. [58] for further details on the deconvolution method.

## Supplementary Information

**Additional file 1: Fig. S1.** The importance of performing hemodynamic deconvolution illustrated for two possible scenarios. (a) The BOLD fMRI signals are highly correlated (the bottom left panel), whereas the latent neural signals are not (the top left panel); (b) the underlying latent neural signals are highly synchronized (the top right panel); however, the correlation between the corresponding BOLD fMRI signals are low (the bottom right panel). Both scenarios result from the fact that the HRFs corresponding to the two signals are not the same and have a delay between them. Therefore, when convolved with the latent neural signals, they can introduce or nullify the shifts in the resulting BOLD signal. The (a) scenario can cause false positives, and (b) scenario lead to false negatives.

## Data Availability

The data used in this study will be made available upon reasonable request to the corresponding author.
