# Supplementary material for: Resting state fMRI connectivity is sensitive to laminar connectional architecture in the human brain
Source: Brain Inform. 2022 Jan 17;9(1):2. doi: 10.1186/s40708-021-00150-4 (PMC8764001; doi:10.1186/s40708-021-00150-4)
Supplement: Supplementary file 1 — Additional file 1: Fig. S1. The importance of performing hemodynamic deconvolution illustrated for two possible scenarios. (a) The BOLD fMRI signals are highly correlated (the bottom left panel), whereas the latent neural signals are not (the top left panel); (b) the underlying latent neural signals are highly synchronized (the top right panel); however, the correlation between the corresponding BOLD fMRI signals are low (the bottom right panel). Both scenarios result from the fact that the HRFs corresponding to the two signals are not the same and have a delay between them. Therefore, when convolved with the latent neural signals, they can introduce or nullify the shifts in the resulting BOLD signal. The (a) scenario can cause false positives, and (b) scenario lead to false negatives. [file 40708_2021_150_MOESM1_ESM.docx]

*
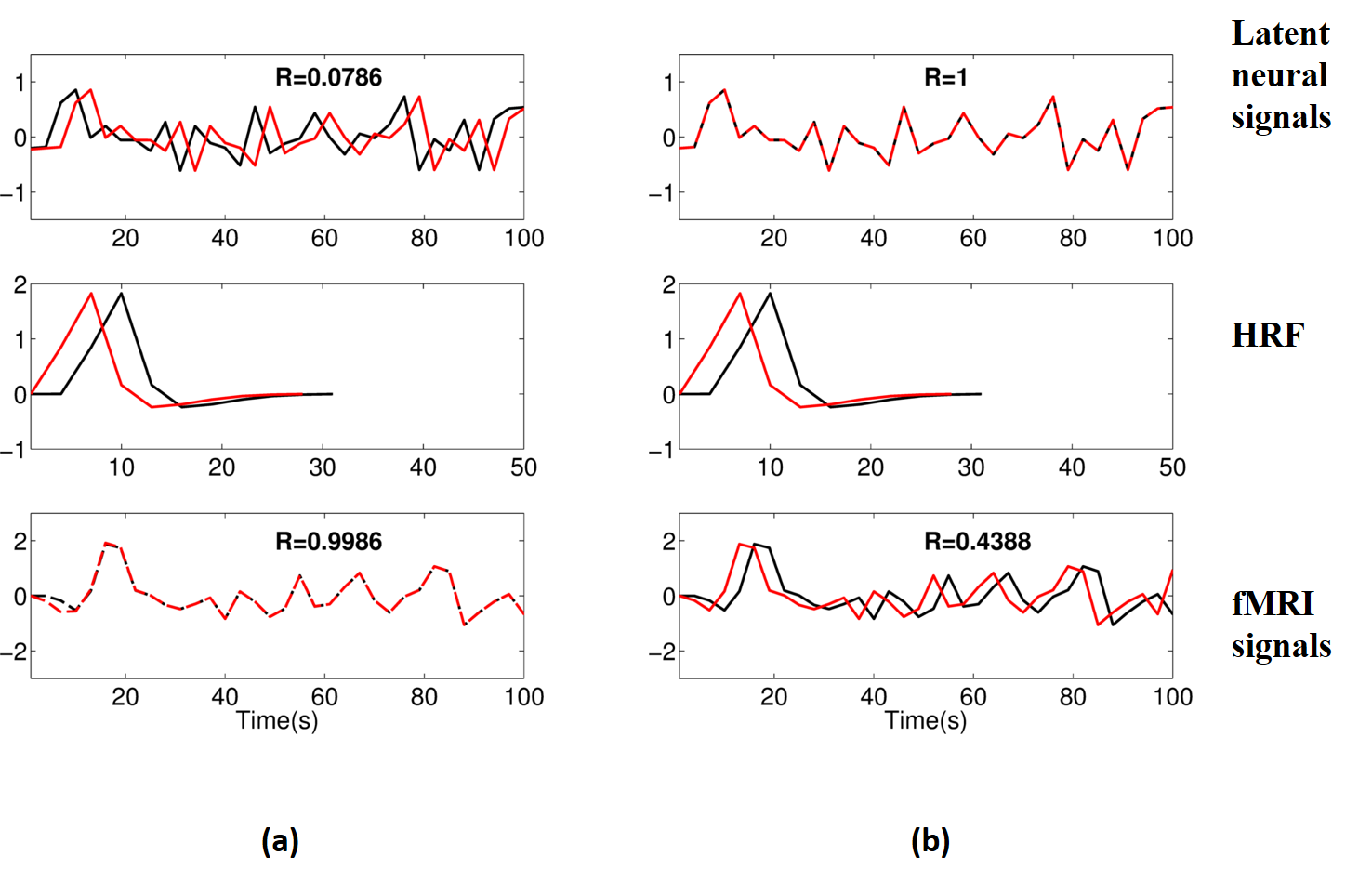
*

**Supplementary Fig. 1.** *The importance of performing hemodynamic deconvolution illustrated for two possible scenarios. (a) The BOLD fMRI signals are highly correlated (the bottom left panel), whereas the latent neural signals are not (the top left panel); (b) the underlying latent neural signals are highly synchronized (the top right panel), however, the correlation between the corresponding BOLD fMRI signals are low (the bottom right panel). Both scenarios result from the fact that the HRFs corresponding to the two signals are not the same and have a delay between them. Therefore, when convolved with the latent neural signals, they can introduce or nullify the shifts in the resulting BOLD signal. The (a) scenario can cause false positives, and (b) scenario lead to false negatives.*
